# SIAH1‐Mediated Ubiquitination and Degradation of TRIB3 Modulate Osteogenic and Adipogenic Differentiation

**DOI:** 10.1155/sci/5507620

**Published:** 2026-08-27

**Authors:** Hao Lv, Hairu Yi, Yinghao Zhu, Yan Wang, Yuxiang Yang, Xingyu Wang, Long Liang, Jiuxiang Wang, Ting Jiang

**Affiliations:** ^1^ Department of Spinal Surgery, The First Affiliated Hospital of Anhui University of Chinese Medicine, Hefei 230000, Anhui Province, China, ahtcm.edu.cn; ^2^ Graduate School, Anhui University of Chinese Medicine, Hefei 230000, Anhui Province, China, ahtcm.edu.cn; ^3^ Anhui Provincial Key Laboratory of Chinese Medicinal Formula, Anhui University of Chinese Medicine, Hefei 230000, Anhui Province, China, ahtcm.edu.cn; ^4^ Clinical Research and Experiment Center, The First Affiliated Hospital of Anhui University of Chinese Medicine, Hefei 230000, Anhui Province, China, ahtcm.edu.cn

## Abstract

**Background:**

The imbalance between osteogenic and adipogenic differentiation of bone marrow mesenchymal stem cells (BMSCs) is a key factor in osteoporosis (OP). TRIB3 acts as a molecular switch regulating this process. While E3 ubiquitin ligases are known to influence TRIB3 protein stability, the specific role of E3 ligase‐mediated TRIB3 ubiquitination in directing BMSCs’ fate remains unclear. This study systematically investigates this mechanism.

**Methods:**

We first predicted potential E3 ubiquitin ligases for TRIB3 using the UbiBrowser database. Differentially expressed E3 ligases were then screened from the GEO datasets. Molecular docking with the TRIB3 protein identified the candidate with the highest binding affinity for a mechanistic study. Serum samples from osteoporotic patients and healthy controls were analyzed to verify clinical correlation. For in vivo validation, we performed in vivo rescue experiments in naturally aged osteoporotic mice using femoral injection of SIAH1‐knockdown lentivirus. Immunohistochemistry, immunofluorescence, and western blot were used to assess the expression of TRIB3 and SIAH1. In vitro, we overexpressed or knocked down SIAH1 to examine its effects on TRIB3 ubiquitination, protein stability, and BMSCs differentiation. TRIB3 rescue assays were performed to confirm the dependency.

**Results:**

UbiBrowser analysis identified eight E3 ubiquitin ligases targeting TRIB3. Subsequent screening selected SIAH1 for mechanistic investigation. In aged osteoporotic mice and osteoporotic patients, SIAH1 expression was significantly increased, while TRIB3 was decreased. In vivo rescue experiments showed that local knockdown of SIAH1 restored TRIB3 expression, improved bone mineral density (BMD), trabecular microarchitecture, and rebalanced osteogenic and adipogenic differentiation. In vitro, upregulating SIAH1 promoted TRIB3 ubiquitination and proteasomal degradation, reducing TRIB3 protein levels, downregulating osteogenic markers, and upregulating adipogenic markers. Knocking down SIAH1 yielded the opposite effects. TRIB3 overexpression or knockdown fully reversed the effects of SIAH1 on BMSC differentiation.

**Conclusion:**

SIAH1 promotes the ubiquitination and proteasomal degradation of TRIB3, thereby disrupting the osteogenic–adipogenic balance of BMSCs and contributing to age‐related bone loss. Local inhibition of SIAH1 stabilizes TRIB3 and ameliorates OP. These findings provide a scientific basis and novel therapeutic target for regulating BMSCs differentiation and treating age‐related OP via the SIAH1–TRIB3 axis.

## 1. Introduction

Osteoporosis (OP) represents a systemic disorder of the skeleton, hallmarked by reduced bone mineral content and the progressive deterioration of trabecular and cortical microarchitecture. Its prevalence rises progressively with chronological age, establishing it as a typical age‐related condition [1]. Available statistical data indicate that among individuals older than 50 years, the diagnosis rate of OP is 6.6% in men versus 22.1% in women, and more than 3 million fragility fractures are recorded each year [2]. The later onset of the disease in men than in women is widely attributed to their comparatively higher androgen levels [3]. In recent years, plant‐based natural products have also received increasing attention as potential therapeutic agents for bone aging and OP [4].

A growing body of evidence has established that disruption of the homeostatic balance between osteogenic and adipogenic lineage commitment in bone marrow mesenchymal stem cells (BMSCs) plays a central role in the pathogenesis of OP [5]. Within this regulatory network, the pseudokinase TRIB3 functions as a pivotal determinant that steers BMSCs toward either osteogenesis or adipogenesis [6–8]. One illustrative example is the ability of TRIB3 to modulate bone morphogenetic protein (BMP) signaling, thereby directly influencing the fate decision of BMSCs [9]. Because the functional stability of the TRIB3 protein is subject to multiple regulatory inputs, post‐translational modifications—especially ubiquitination followed by proteasomal degradation—have garnered considerable interest. Notably, multiple lines of evidence, including reviews on plant‐derived compounds, have highlighted the importance of maintaining the osteogenic–adipogenic balance for bone health [4]. Although E3 ubiquitin ligases are the key arbiters of substrate specificity in the ubiquitination cascade, it remains unknown which E3 ligase specifically targets TRIB3 and how this event regulates BMSC differentiation in the context of OP.

This study, therefore, aims to elucidate the regulatory network of TRIB3 ubiquitination and its functional mechanism in BMSC differentiation. We employed bioinformatics analysis to predict E3 ligases interacting with TRIB3. Using both in vivo and in vitro experiments, we then verify the effect of E3 ligases on TRIB3 ubiquitination and its subsequent impact on osteogenic and adipogenic differentiation.

## 2. Materials and Methods

### 2.1. Bioinformatics Screening

Potential E3 ligases for TRIB3 were predicted using UbiBrowser 2.0 (http://ubibrowser.bio-it.cn/ubibrowser_v3/). Differentially expressed E3 ligases were then identified from the GEO dataset GSE230665. Finally, protein–protein docking between the candidate E3 ligases and TRIB3 was performed using the Scicompute platform (http://www.scicompute.com/) to select the E3 ubiquitin ligase with the strongest predicted binding affinity for further mechanistic investigation.

### 2.2. Experimental Validation

#### 2.2.1. Experimental Animals and Materials

Thirty SPF‐grade mice were purchased from Hangzhou Ziyuan Laboratory Animal Technology Co., Ltd. (Production License No.: SCXK (ZHE) 2019‐0004; Animal Qualification Certificate No.: 20240624Abzz0105000251). These mice were housed at the Animal Experiment Center of the First Affiliated Hospital of Anhui University of Chinese Medicine under controlled conditions (20–25°C, 45%–60% humidity, and 12‐h light/dark cycle) with free access to food and water following a 1‐week acclimation period.

The experimental protocol was approved by the Animal Experiment Ethics Committee of the First Affiliated Hospital of Anhui University of Chinese Medicine (Approval No.: AZYFY‐2024‐2006) and conducted in accordance with the “Consensus Author Guidelines for Animal Ethics and Welfare” and relevant regulations.

Human bone marrow samples were collected from patients undergoing orthopedic surgery at the First Affiliated Hospital of Anhui University of Chinese Medicine with written informed consent. The study was approved by the hospital’s Medical Ethics Committee (Approval No.: 2024AH‐143).

#### 2.2.2. Experimental Instruments

The following instruments were used: EPS‐300 electrophoresis apparatus (Tanon); chemiluminescence imaging system (Biorad); CO_2_ incubator (NuAire); Multiskan spectrophotometer (BioTek Instruments); flow cytometer (Beckman Coulter); high‐speed centrifuge (Eppendorf); western blot system (Tanon Science & Technology); ice maker (Xueke Electric); inverted microscope (Olympus); cell counter (Countstar); clean bench (AIRTECH); and cell culture plates and flasks (Corning).

#### 2.2.3. Reagents and Antibodies

The following commercially available enzyme‐linked immunosorbent assay (ELISA) kits were used for the quantification of serum SIAH1 and TRIB3 protein levels. The SIAH1 ELISA kit (catalog No. AF0334559‐A) and the TRIB3 ELISA kit (catalog No. AF0334560‐A) were both purchased from Aifang Biological Technology Co., Ltd.

Reagents included sodium pentobarbital (Sigma); paraformaldehyde (Biosharp); column total protein extraction kit and ultra‐sensitive ECL development kit (Saiwen Innovation); high‐glucose DMEM (HG‐DMEM) and fetal bovine serum (Gibco); 0.25% trypsin–EDTA, penicillin–streptomycin, and PBS (Hyclone); osteogenic and adipogenic induction differentiation kits, alizarin red S, and oil red O staining solutions (Cyagen); alkaline phosphatase (ALP) chromogenic kit and immunoprecipitation kit (Beyotime); and cycloheximide (CHX) and MG132 (MedChemExpress).

Primary antibodies were used against the following proteins at the indicated dilutions: SIAH1 (HUABIO, HA721363, 1:1000), TRIB3 (Biodragon, BD‐PN1887, 1:1000), ALP (Biodragon, RM9006, 1:1000), RUNX2 (Biodragon, RM8028, 1:1000), PPARγ (Biodragon, RM5076, 1:1000), adiponectin (BOSTER, M00509‐4, 1:1000), GAPDH (BOSTER, BA2913, 1:10000), CD34, and CD90 (Abcam).

The SIAH1 overexpression plasmid and Flag‐TRIB3 plasmid were constructed by the MiaoLing Plasmid Platform, and SIAH1 siRNA was purchased from Hippo Bio.

### 2.3. Measurement of Serum E3 Ubiquitin Ligase and TRIB3 Protein Levels in Osteoporotic Patients and Nonosteoporotic Controls

Serum levels of E3 ubiquitin ligase and TRIB3 in OP and non‐OP participants were determined using ELISA kits specifically targeting these proteins. Peripheral blood samples were collected from 30 patients with OP and 30 age‐matched healthy controls (non‐OP) at the First Affiliated Hospital of Anhui University of Chinese Medicine. Written informed consent was obtained from all participants. The study was approved by the hospital’s Medical Ethics Committee (Approval No. 2024AH‐143). Blood was drawn from the antecubital vein into vacuum tubes without an anticoagulant and allowed to clot at room temperature for 30 min. Serum was separated by centrifugation at 3000 g for 15 min at 4°C and then aliquoted and stored at −80°C until analysis. Repeated freeze–thaw cycles were avoided. All assays were performed according to the manufacturer’s protocols. Briefly, serum samples were thawed on ice and diluted with the provided sample diluent, as recommended. Standards and samples (100 µL per well) were added in duplicate to the pre‐coated 96‐well plates and incubated at 37°C for 90 min. After discarding the liquid, 100 µL of biotinylated detection antibody working solution was added to each well and incubated at 37°C for 60 min. The plates were then washed three times with wash buffer, followed by the addition of 100 µL of horseradish peroxidase (HRP)‐conjugated streptavidin working solution and incubation at 37°C for 30 min in the dark. After another washing step, 90 µL of tetramethylbenzidine (TMB) substrate solution was added per well and incubated at 37°C for 15–30 min in the dark. The reaction was stopped by adding 50 µL of a stop solution (2 M H_2_SO_4_). Absorbance was read at 450 nm using a microplate reader (BioTek Instruments, Winooski, VT, USA). Protein concentrations were calculated from the standard curves generated with the kit standards.

### 2.4. In Vivo Studies

#### 2.4.1. Experimental Grouping

Three‐month‐old C57BL/6 mice served as the control group, while 20‐month‐old mice constituted the natural aging osteoporotic model group, of which 10 20‐month‐old mice served as the model group and 10 20‐month‐old mice served as the SIAH1 knockdown group. Mice in the SIAH1 lentivirus knockdown group were anesthetized with 1% sodium pentobarbital (20 mg/kg, i.p.). Under sterile conditions, a 26‐gauge microsyringe was inserted into the distal femoral metaphysis through the patellar tendon. A volume of 10 µL of lentivirus (1 × 10^8^ TU/mL) was slowly injected into the bone marrow cavity of each femur. The injection was performed bilaterally. Mice were allowed to recover and were euthanized 6 weeks after injection.

#### 2.4.2. Micro‐CT Analysis of Bone Morphology

Following a 12‐h fast, mice were anesthetized via an intraperitoneal injection of 1% sodium pentobarbital (20 mg/kg) and euthanized by cervical dislocation. The surrounding muscle tissue was dissected from the femur, which was then fixed in 4% paraformaldehyde. The left femur was scanned using a high‐resolution micro‐CT imaging system (Imaging 100, Raycision Medical Technology Co., Ltd.). Quantitative analysis of the images yielded parameters including bone mineral density (BMD, mg/cm^3^), bone volume/tissue volume (BV/TV, %), trabecular thickness (Tb.Th, mm), trabecular number (Tb.N, 1/mm), and trabecular separation (Tb.Sp, mm). The reconstructed micro‐CT data were visualized three‐dimensionally using 3D Slicer software to capture representative images.

#### 2.4.3. Serum Biochemical Indicators

Serum levels of ALP, Ca, and P were measured. After collection, serum samples were stored overnight at 2–8°C and centrifuged to obtain the supernatant, with care taken to avoid repeated freeze–thaw cycles. Appropriate anticoagulants were selected based on the specific analyte.

#### 2.4.4. HE Staining

Mouse bone tissue was fixed in 4% paraformaldehyde for 24 h and decalcified in 10% EDTA at 4°C. The decalcification solution was changed every 3 days, and completion was confirmed when a pin could easily penetrate the bone. The softened bone blocks were rinsed with distilled water, dehydrated through an ethanol series, cleared, infiltrated with wax, and embedded in paraffin. Sections of 4 μm thickness were cut, stained conventionally with hematoxylin and eosin, dehydrated, mounted, and examined microscopically to assess trabecular morphology.

#### 2.4.5. TRAP Staining

Tissue sections were dewaxed and rehydrated. After incubation in pure water at 37°C for 2 h, sections were stained with the TRAP staining solution, dehydrated, mounted, and imaged for analysis.

#### 2.4.6. Masson Staining

Following dewaxing and rehydration, sections were stained with Masson dye, counterstained, dehydrated, cleared, and mounted with neutral resin for microscopic observation and image analysis.

#### 2.4.7. Immunofluorescence

Paraffin sections of femoral tissue were dewaxed, rehydrated, and subjected to antigen retrieval. Sections were blocked with 3% bovine serum albumin for 30 min at room temperature and then incubated overnight at 4°C with fluorescently labeled primary antibodies against SIAH1 and TRIB3 in a humidified, light‐protected chamber. Cell nuclei were counterstained with DAPI and sections were mounted with an anti‐fade medium before imaging with an inverted fluorescence microscope.

#### 2.4.8. Immunohistochemistry

Tissue samples were fixed, processed, embedded in paraffin, and sectioned. Sections were baked, cooled, and treated with 3% hydrogen peroxide to quench endogenous peroxidase activity. After washing with PBS, nonspecific binding was blocked with 5% normal goat serum. Sections were incubated overnight at 4°C with primary antibodies against SIAH1 and TRIB3 (1:200 dilution). Following PBS washes, sections were incubated with biotinylated goat anti‐rabbit IgG (1:200) and then with HRP–streptavidin complex. Color development was performed with DAB and the reaction was stopped by rinsing with water. After counterstaining with hematoxylin, dehydration, and mounting, sections were examined and photographed under a microscope.

#### 2.4.9. Western Blot Analysis

Proteins were extracted following the kit instructions, separated by electrophoresis, and transferred to a membrane. After blocking, membranes were incubated with primary antibodies overnight at 4°C and with secondary antibodies for 2 h at room temperature. Bands were visualized by chemiluminescence and their intensity was quantified using ImageJ software. Target protein expression levels were normalized to GAPDH.

### 2.5. In Vitro Studies

#### 2.5.1. Cell Isolation, Culture, and Identification

Human BMSCs (hBMSCs) were isolated and cultured via the whole bone marrow adherence method, as previously described [10]. Bone marrow tissue fragments were placed in HG‐DMEM supplemented with 10% fetal bovine serum, 100 U/mL penicillin, and 100 µg/mL streptomycin. The medium was refreshed every 2–3 days. Upon reaching confluence, cells were detached with trypsin, centrifuged, and passaged into T25 flasks. Cells from passages 3–5 were used for all subsequent experiments.

#### 2.5.2. Cell Phenotype Identification by Flow Cytometry

Fourth‐passage cells were digested with 0.25% trypsin for 1 min and centrifuged at 1000 × *g* for 5 min. After discarding the supernatant, the pellet was resuspended and incubated with CD34/CD90 antibodies for 30 min in the dark. Phenotypic expression was then analyzed by flow cytometry.

#### 2.5.3. Cell Transfection

Fourth‐passage cells were trypsinized, centrifuged at 1000 × *g* for 5 min, and resuspended in 1 mL of fresh medium for counting. Cells were seeded at a density of 1 × 10^5^ cells per well. Twenty‐four h later, transfection was performed using the Lipofectamine Stem Cell Transfection Reagent kit according to the manufacturer’s protocol. After 6–8 h, the medium was replaced with a fresh antibiotic‐free medium. Cells were harvested 72 h post‐transfection for protein detection or staining.

#### 2.5.4. Western Blot Analysis

Western blotting for in vitro samples was performed using the same protocol as that described above for the in vivo study. Briefly, proteins were extracted, separated by electrophoresis, transferred to membranes, and incubated with the indicated primary antibodies, followed by HRP‐conjugated secondary antibodies. Protein bands were visualized by chemiluminescence and quantified using ImageJ software.

#### 2.5.5. CHX Chase Assay

To evaluate the effect of SIAH1 on TRIB3 protein stability, cells transfected with either a negative control (NC) or SIAH1‐overexpressing (OE SIAH1) plasmid were treated with 100 µg/mL CHX. Cells from each group were collected at 0, 2, 4, 6, and 8 h after CHX addition to monitor TRIB3 degradation over time.

#### 2.5.6. MG132 Proteasome Inhibition Assay

To examine proteasome pathway involvement, cells were assigned to four groups: NC, OE SIAH1, OE SIAH1 with DMSO (vehicle), and OE SIAH1 with the proteasome inhibitor MG132. At 48 h post‐transfection, the respective groups were treated with 10 µM MG132 or an equivalent volume of DMSO for 6 h before collection.

#### 2.5.7. ALP Staining

Third‐ to fifth‐passage cells were seeded in six‐well plates at 1 × 10^5^ cells per well and transfected with NC, OE SIAH1, or SIAH1 siRNA constructs using Lipofectamine Stem Cell Transfection Reagent. Seventy‐two hours later, the medium was replaced with an osteogenic induction medium. Cells were maintained in a 37°C, 5% CO_2_ incubator for 2 weeks, with medium changes every 48 h. Parallel wells were then stained according to the kit protocol, and images were acquired.

#### 2.5.8. Alizarin Red S Staining

Cells from passages 3–5 were plated in six‐well plates. After 72 h, the medium was switched to an osteogenic induction medium and cultures were maintained for 2 weeks under standard conditions. Parallel wells were stained with alizarin red S and the results were documented.

#### 2.5.9. Oil Red O Staining

Third‐passage cells were seeded in six‐well plates. Seventy‐two hours later, adipogenic induction medium was applied and cells were cultured for 2 weeks. Cells were then stained using an oil red O kit to visualize intracellular lipid droplets. To elute the bound dye, 1 mL of 100% isopropanol was added and incubated at 37°C for 10 min.

### 2.6. Statistical Analysis

Data were analyzed with SPSS 27.0 and graphed using GraphPad Prism 9. Continuous variables are expressed as mean ± standard deviation ($\bar{x}\pm s$). For data satisfying assumptions of normality and homogeneity of variance, two‐group comparisons employed the independent sample *t*‐test, while multiple‐group comparisons used one‐way ANOVA. Nonparametric alternatives (Mann–Whitney *U* test for two groups and Kruskal–Wallis *H* test for multiple groups) were applied otherwise. Categorical variables were analyzed with the *χ* ^2^ test. A *p*‐value < 0.05 was considered statistically significant.

## 3. Results

### 3.1. Bioinformatics Screening

**Figure 1:**
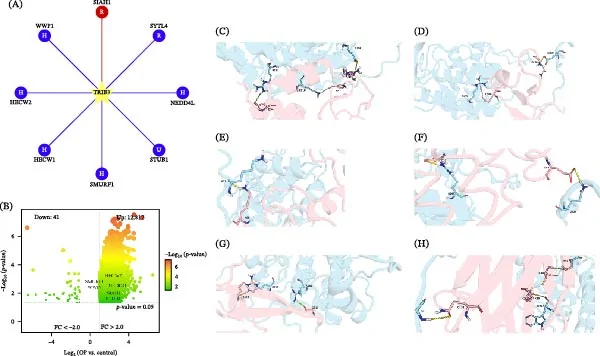
Bioinformatics screening of E3 ubiquitin ligases targeting TRIB3. (A) UbiBrowser 2.0 prediction of eight potential E3 ubiquitin ligases (SIAH1, SYTL4, NEDD4L, STUB1, SMURF1, HECW2, WWP1, and HECW1) that may target TRIB3. (B) Identification of six differentially expressed E3 ligases (SIAH1, NEDD4L, STUB1, SMURF1, HECW2, and WWP1) from the GEO dataset GSE230665. (C–H) Protein‐protein docking visualization of the six candidate E3 ligases with TRIB3.

UbiBrowser 2.0 predicted eight E3 ligases targeting TRIB3 (SIAH1, SYTL4, NEDD4L, STUB1, SMURF1, HECW2, WWP1, and HECW1) (Figure 1A). Subsequently, six differentially expressed E3 ligases were identified in the GEO dataset (GSE230665): SIAH1, NEDD4L, STUB1, SMURF1, HECW2, and WWP1 (Figure 1B). These six E3 ligases were subjected to protein–protein docking with the TRIB3 protein on the http://www.scicompute.com/ website. The resulting docking energies are provided in Supporting Information 1: File 1, and the visualization results are shown in Figure 1C–H. The results indicate that SIAH1 demonstrated the strongest predicted binding affinity to TRIB3.

### 3.2. Serum Levels of E3 Ubiquitin Ligase (SIAH1) and TRIB3 in OP and Non‐OP Participants

**Figure 2:**
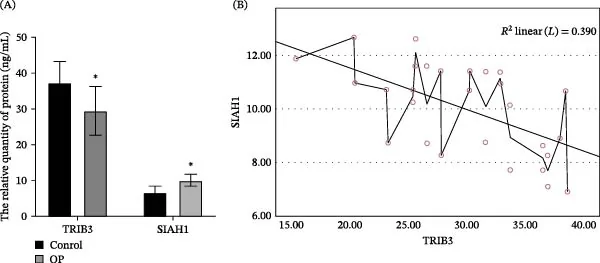
Serum expression and correlation analysis of SIAH1 and TRIB3. (A) Serum levels of SIAH1 and TRIB3 in OP patients (*n* = 30) and non‐OP controls (*n* = 30) measured by ELISA.  ^∗^ *p* < 0.05 vs. control. (B) Linear regression analysis between serum SIAH1 and TRIB3 levels.

To further validate the association between SIAH1 and TRIB3, serum samples were collected from 30 patients with OP and 30 age‐matched non‐OP controls. The expression levels of SIAH1 and TRIB3 were measured by ELISA and a correlation analysis between the two proteins was performed. As shown in Figure 2A, compared with the non‐OP control group, the OP group exhibited significantly higher serum levels of SIAH1 (*p* < 0.05) and significantly lower levels of TRIB3 (*p* < 0.05). Linear regression analysis revealed a strong negative correlation between SIAH1 and TRIB3 levels, with an *R* ^2^ value of 0.39 (*p* < 0.01) (Figure 2B). Spearman correlation analysis further confirmed a significant negative association between the two proteins (Spearman’s *r* = −0.641, *p*  < 0.001). These results indicate that elevated SIAH1 is closely associated with reduced TRIB3 in the serum of osteoporotic patients.

### 3.3. In Vivo Studies

#### 3.3.1. Analysis of Micro‐CT, Bone Parameters, and Serum Biochemistry

**Figure 3:**
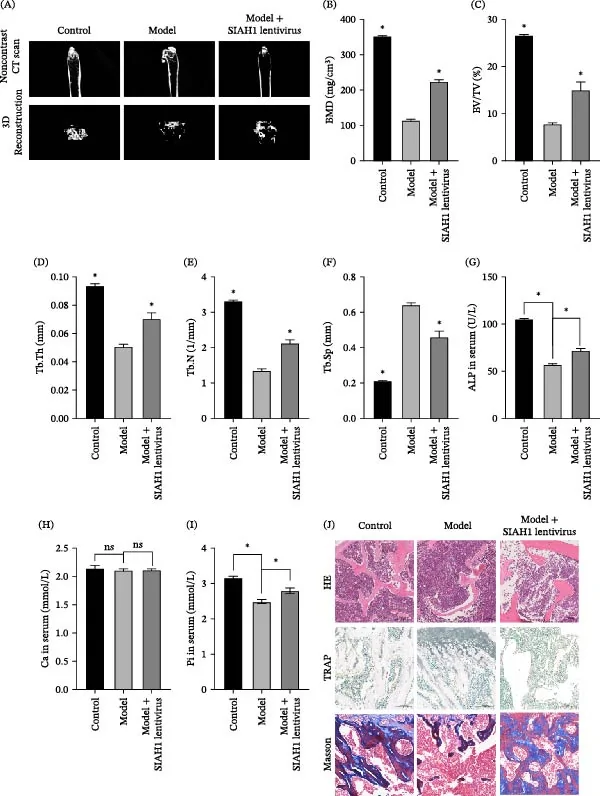
It presents the results of micro‐CT scanning, bone parameters, serum biochemical indices, and tissue staining for the control, model, and model + SIAH1 Lentivirus group mice. (A) Representative micro‐CT three‐dimensional reconstruction images of distal femurs. (B–F) Quantitative analysis of BMD, BV/TV, Tb.Th, Tb. N, and Tb.Sp (*n* = 6 per group). (G–I) Serum levels of ALP, Ca, and Pi (*n* = 6 per group). (J) Representative images of HE staining, TRAP staining, and Masson staining of femoral sections (scale bar = 100 μm).  ^∗^ *p* < 0.05 vs. model; ^#^ *p* < 0.05 vs. control. ns, not significant.

Micro‐CT three‐dimensional reconstruction showed that the control group had relatively uniform trabecular bone distribution, slightly coarse morphology, and dense cortical bone. In contrast, the model group exhibited substantial trabecular breakage, a marked reduction in Tb.N, and noticeably thinner cortical bone. Notably, the model + SIAH1 Lentivirus group showed significant improvement in trabecular structure, with more numerous and better‐connected trabeculae compared with the model group (Figure 3A).

Quantitative analysis of bone parameters revealed that, compared with the control group, the model group had significantly lower BMD, BV/TV, Tb. Th, and Tb. N, and significantly higher Tb.Sp (all *p* < 0.05). Importantly, SIAH1 knockdown significantly reversed these changes. The model + SIAH1 Lentivirus group exhibited higher BMD, BV/TV, Tb. Th, and Tb. N, and lower Tb. Sp compared with the model group (all *p* < 0.05) (Figure 3B–F). However, despite the significant improvement, the bone microstructural parameters (BMD, BV/TV, Tb. N, Tb.Th, and Tb.Sp) in the Model + SIAH1 Lentivirus group did not fully normalize to the levels observed in the control group.

Serum biochemical analysis showed that ALP and inorganic phosphorus (Pi) levels were significantly decreased in the Model group compared with the control group, while calcium (Ca) levels showed no significant difference. SIAH1 knockdown partially restored ALP and Pi levels, although they remained lower than those in the control group (Figure 3G–I).

#### 3.3.2. Analysis of Tissue Staining

HE staining demonstrated that trabecular bone in the control group was relatively coarse and regularly arranged. The Model group exhibited sparse and fewer trabeculae, whereas the model + SIAH1 Lentivirus group showed thicker and more continuous trabeculae with improved connectivity (Figure 3J).

TRAP staining revealed an increased number of osteoclasts in the model group compared with the control group. In contrast, the model + SIAH1 Lentivirus group had fewer osteoclasts on the bone surface, indicating reduced bone resorption (Figure 3J).

Masson staining showed intact and orderly connected trabecular bone with visible light‐blue epiphyseal growth plates in control mice. The model group displayed sparse and fractured trabeculae with broken connections and thinner growth plates. SIAH1 knockdown ameliorated these abnormalities, as evidenced by more intact collagen fibers and a better‐preserved bone matrix (Figure 3J).

Histological analysis further confirmed that SIAH1 knockdown alleviated the osteoporotic phenotype, but the trabecular architecture, osteoclast number, and collagen organization in the model + SIAH1 Lentivirus group did not completely recover to the control levels.

#### 3.3.3. Analysis of Immunofluorescence and Immunohistochemical Results

**Figure 4:**
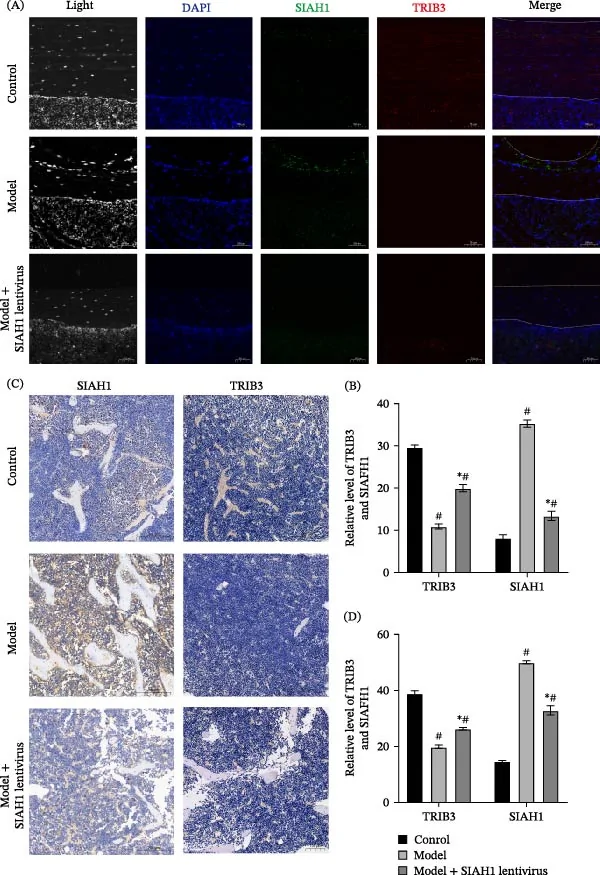
Immunofluorescence and immunohistochemical detection results of SIAH1/TRIB3 in the femoral bone tissue of the control, model, and model + SIAH1 Lentivirus group mice. (A) Representative immunofluorescence images of SIAH1 (green) and TRIB3 (red) in femoral sections. Nuclei were counterstained with DAPI (blue). Scale bar = 50 μm. (B) Quantification of fluorescence intensity. (C) Representative immunohistochemistry images of SIAH1 and TRIB3. Scale bar = 200 μm. (D) Quantification of immunohistochemical staining intensity.  ^∗^ *p* < 0.05 vs. model; ^#^ *p* < 0.05 vs. control.

Immunofluorescence staining of femoral bone sections showed that SIAH1 fluorescence intensity was significantly higher and TRIB3 intensity significantly lower in the model group compared with the control group. In the model + SIAH1 Lentivirus group, SIAH1 expression was markedly reduced, while TRIB3 expression was partially restored compared with the model group (Figure 4A,B).

Correspondingly, immunohistochemical analysis confirmed elevated SIAH1 protein expression and reduced TRIB3 expression in the Model group relative to controls. SIAH1 knockdown significantly decreased SIAH1 levels and increased TRIB3 levels (Figure 4C,D).

Although SIAH1 knockdown significantly reduced SIAH1 expression and partially restored TRIB3 expression, the fluorescence intensity and immunohistochemical staining signals of both proteins in the model + SIAH1 Lentivirus group did not reach the levels seen in the control group.

#### 3.3.4. Analysis of Western Blot Results

**Figure 5:**
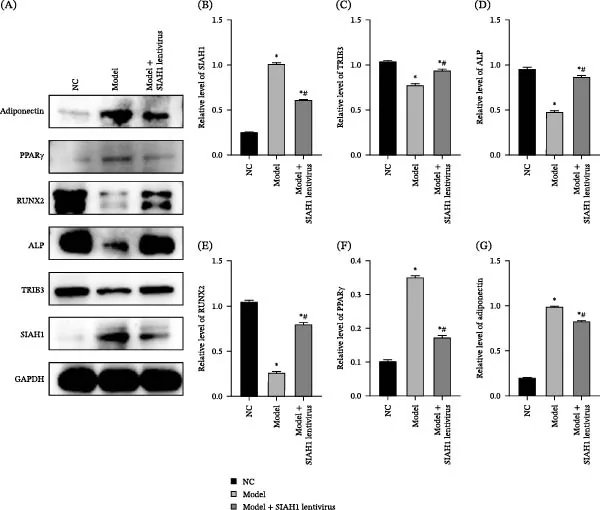
Western blot results of SIAH1/TRIB3 in the femoral bone tissue of the control, model, and model + SIAH1 Lentivirus group mice. Representative Western blot bands and quantitative analysis of protein levels (*n* = 3 per group). GAPDH was used as a loading control.  ^∗^ *p* < 0.05 vs. control; ^#^ *p* < 0.05 vs. model. (A) Representative western blot bands for SIAH1, TRIB3, ALP, RUNX2, PPARγ, adiponectin, and GAPDH (loading control). (B–G) Quantitative analysis of protein expression levels (normalized to GAPDH) for SIAH1 (B), TRIB3 (C), ALP (D), RUNX2 (E), PPARγ (F), and adiponectin (G), respectively.

As shown in Figure 5, compared with the control group, the model group exhibited a significant increase in SIAH1 expression (*p* < 0.01) and a marked decrease in TRIB3 expression (*p* < 0.01). Correspondingly, the expression levels of osteogenic markers ALP and RUNX2 were significantly reduced in the model group, while the adipogenic markers PPARγ and adiponectin were significantly elevated (all *p* < 0.05 vs. control). Remarkably, lentivirus‐mediated SIAH1 knockdown in the model + SIAH1 Lentivirus group reversed these changes. Compared with the model group, SIAH1 protein levels were substantially reduced (*p* < 0.05), and TRIB3 levels were restored (*p* < 0.05). Furthermore, SIAH1 knockdown led to a significant upregulation of ALP and RUNX2, and a marked downregulation of PPARγ and adiponectin (all *p* < 0.05 vs. model). These results indicate that in vivo knockdown of SIAH1 not only restores TRIB3 expression but also shifts the differentiation balance of BMSCs toward osteogenesis and away from adipogenesis in aged osteoporotic mice.

However, despite these improvements, the expression levels of SIAH1, TRIB3, and the differentiation markers in the Model + SIAH1 Lentivirus group did not fully return to those observed in the control group.

### 3.4. In Vitro Studies

#### 3.4.1. Effect of Modulating SIAH1 Expression on TRIB3 and Osteogenic/Adipogenic Marker Expression

**Figure 6:**
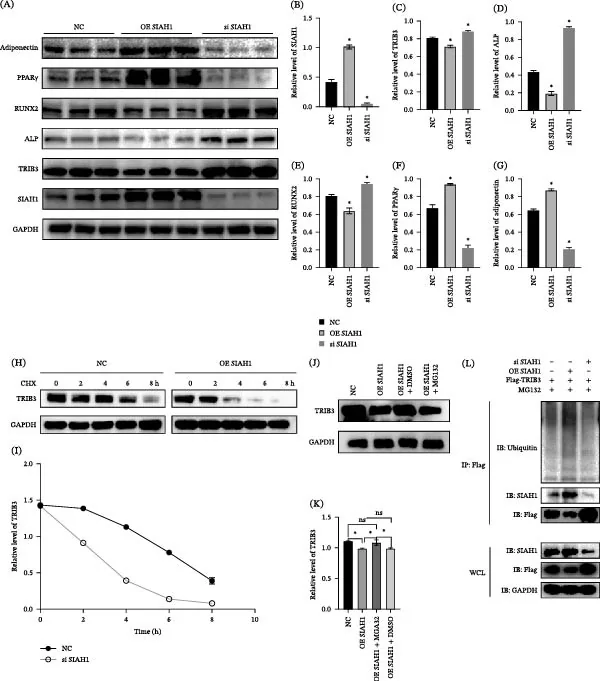
The effect of SIAH1 on TRIB3 protein ubiquitination and osteogenic/adipogenic differentiation. (A–G) Western blot analysis of SIAH1, TRIB3, ALP, RUNX2, PPARγ, and adiponectin in BMSCs transfected with NC, OE SIAH1, or si SIAH1. (H, I) CHX chase assay showing the half‐life of TRIB3 in NC and OE SIAH1 groups. (J, K) MG132 proteasome inhibition assay. (L) Co‐IP and IP‐WB analysis of TRIB3 ubiquitination in BMSCs co‐transfected with Flag‐TRIB3 and either OE SIAH1 or si SIAH1.  ^∗^ *p* < 0.05. ns, not significant.

As shown in Supporting Information 2: File 2, all primary cell cultures were used at 3–5 passages, and P4 cells were detected with flow cytometry. Transfection of BMSCs with an SIAH1 plasmid significantly increased the SIAH1 protein levels. In the SIAH1 overexpression group (OE SIAH1), expression of TRIB3 and the osteogenic markers ALP and RUNX2 decreased, whereas levels of the adipogenic markers Adiponectin and PPARγ increased. Conversely, transfection with the SIAH1 siRNA markedly reduced SIAH1 expression. In this knockdown group (si SIAH1), TRIB3, ALP, and RUNX2 levels were significantly elevated, while adiponectin and PPARγ levels were reduced (Figure 6A–G).

#### 3.4.2. Effect of SIAH1 on TRIB3 Protein Stability

After CHX treatment, the half‐life of the TRIB3 protein was significantly shorter in the OE SIAH1 group (Figure 6H,I). Treatment with the proteasome inhibitor MG132 substantially increased TRIB3 protein levels compared to the OE SIAH1 group and the OE SIAH1 + DMSO control, reversing the downregulation caused by SIAH1 overexpression (Figure 6J,K). These results demonstrate that SIAH1 regulates TRIB3 through the ubiquitin‐proteasome pathway.

#### 3.4.3. Effect of SIAH1 on TRIB3 Ubiquitination

We next investigated whether SIAH1 directly mediates TRIB3 ubiquitination. BMSCs were co‐transfected with Flag‐TRIB3 and either SIAH1 overexpression or knockdown constructs. Co‐IP confirmed an interaction between SIAH1 and TRIB3. IP‐WB analysis targeting ubiquitin revealed that TRIB3 ubiquitination increased with SIAH1 overexpression and decreased with SIAH1 knockdown (Figure 6L).

#### 3.4.4. TRIB3 Rescue Reverses the Effects of SIAH1 on Osteogenic and Adipogenic Marker Expression

To determine whether SIAH1 regulates BMSC differentiation specifically through TRIB3, rescue experiments were performed. BMSCs were divided into five groups: NC, SIAH1 overexpression alone (OE SIAH1), SIAH1 overexpression combined with TRIB3 overexpression (OE SIAH1 + OE TRIB3), SIAH1 knockdown alone (si SIAH1), and SIAH1 knockdown combined with TRIB3 knockdown (si SIAH1 + si TRIB3). Protein expression levels of SIAH1, TRIB3, osteogenic markers (ALP and RUNX2), and adipogenic markers (PPARγ and adiponectin) were assessed by western blot.

**Figure 7:**
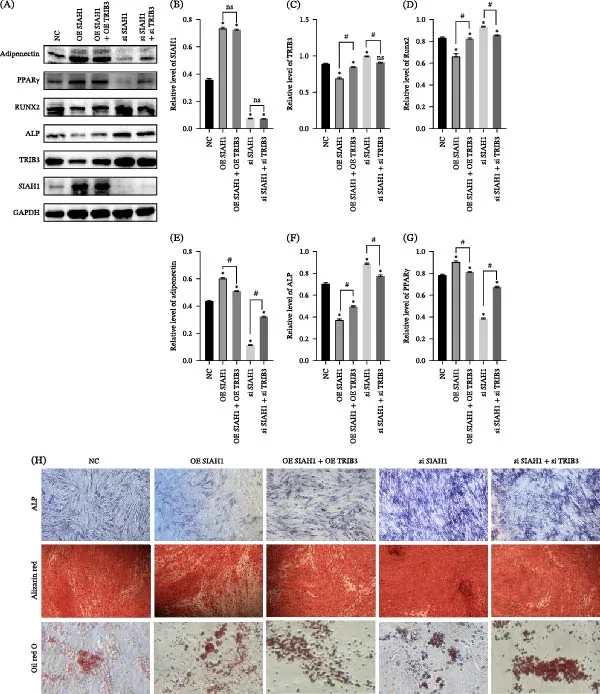
TRIB3 restoration reverses SIAH1‐induced alterations in osteogenic and adipogenic markers and cell staining. (A–G) Western blot analysis of SIAH1, TRIB3, ALP, RUNX2, PPARγ, and adiponectin in BMSCs from five groups: NC, OE SIAH1, OE SIAH1 + OE TRIB3, si SIAH1, and si SIAH1 + si TRIB3. (H) Representative images of ALP staining, alizarin red S staining, and oil red O staining.  ^∗^ *p*  < 0.05 vs. NC. ^#^ *p* < 0.05 vs. OE SIAH1 or si SIAH1 as indicated.

As shown in Figure 7A–G, OE SIAH1 alone significantly increased SIAH1 expression (*p* < 0.01 vs. NC) and decreased TRIB3, ALP, and RUNX2, while increasing PPARγ and Adiponectin (all *p* < 0.05 vs. NC). Notably, co‐overexpression of TRIB3 (OE SIAH1 + OE TRIB3) did not alter the elevated SIAH1 levels but significantly restored TRIB3 expression (*p* < 0.05 vs. OE SIAH1). Consequently, the reduced osteogenic markers and elevated adipogenic markers caused by SIAH1 overexpression were markedly reversed by TRIB3 co‐overexpression (all *p* < 0.05 vs. OE SIAH1). Conversely, si SIAH1 alone significantly reduced SIAH1 expression (*p* < 0.01 vs. NC) and increased TRIB3, ALP, and RUNX2, while decreasing PPARγ and Adiponectin (all *p* < 0.05 vs. NC). Co‐knockdown of TRIB3 (si SIAH1 + si TRIB3) did not affect the reduced SIAH1 levels but effectively suppressed the si SIAH1‐induced increase in TRIB3 (*p* < 0.05 vs. si SIAH1). As a result, the enhanced osteogenic markers and reduced adipogenic markers observed upon SIAH1 knockdown were largely reversed by TRIB3 co‐knockdown (all *p* < 0.05 vs. si SIAH1). These results confirm that the regulatory effects of SIAH1 on osteogenic and adipogenic differentiation are mediated via TRIB3.

#### 3.4.5. Analysis of Cell Staining Results

Consistent with the Western blot results, cell staining assays further validated the rescue effects (Figure 7H). ALP staining and alizarin red S staining showed that the decreased calcium deposition and calcified nodule formation induced by SIAH1 overexpression were markedly restored by co‐expression of TRIB3. Conversely, the increased mineralization observed upon SIAH1 knockdown was reversed by simultaneous TRIB3 knockdown. Oil red O staining demonstrated that the enhanced lipid droplet accumulation caused by SIAH1 overexpression was attenuated by TRIB3 overexpression, whereas the reduced adipogenesis in siSIAH1‐treated cells was rescued by TRIB3 knockdown. These findings collectively indicate that SIAH1 modulates the osteogenic/adipogenic differentiation of BMSCs through the targeted regulation of TRIB3.

## 4. Discussion

Protein ubiquitination is a crucial post‐translational modification that secures protein homeostasis and proper cellular functioning through the ubiquitin‐proteasome pathway. This process involves a hierarchical cascade of E1 (activating), E2 (conjugating), and E3 (ligase) enzymes, with the E3 ligase dictating substrate specificity [11–16]. Numerous E3 ligases—such as NEDD4L, STUB1, SMURF1, and WWP1—have been implicated in osteogenic differentiation by promoting the degradation of key osteogenic transcription factors, including RUNX2 and BMAL1 [17–20]. In parallel, TRIB3 serves as an essential pseudokinase that governs the balance between osteogenesis and adipogenesis in BMSCs; its own stability is likewise controlled by E3 ligase‐mediated ubiquitination [21]. However, the specific E3 ligase that mediates TRIB3 ubiquitination and regulates BMSCs’ fate determination in OP remained unclear. Through bioinformatic prediction, molecular docking, and experimental validation, we identified SIAH1 as the bona fide E3 ligase that directly binds and ubiquitinates TRIB3 to promote its proteasomal degradation.

Notably, a prior study reported osteopenia in Siah1a mutant mice, which is fully consistent with our present findings [22]. However, the underlying mechanism linking SIAH1 deficiency to bone loss was not explored in this study. SIAH1 is a 32 kDa E3 ubiquitin ligase encoded on chromosome 16q13, featuring an N‐terminal RING zinc finger domain for E2 binding and a C‐terminal substrate‐binding domain [23, 24]. It directly binds TRIB3 to promote its K48‐linked ubiquitination and shorten its half‐life. While earlier studies have established that the E3 ubiquitin ligase SIAH1 can ubiquitinate and degrade TRIB3 in cancer or heterologous cells [12, 23, 25], the functional role of this regulatory axis in determining the lineage fate of BMSCs, specifically the balance between osteogenic and adipogenic differentiation, has remained unexplored. Several recent studies have advanced our understanding of BMSC differentiation and bone metabolism but through distinct mechanisms. Ni et al. [26] identified a lncRNA‐miRNA‐PAX8 axis that promotes osteogenesis, while Zhang et al. [27] showed that 4,4‐dimethylsterols reduce fat accumulation via the inhibition of fatty acid amide hydrolase. Wu and Sun [28] demonstrated functional redundancy among neddylation E2/E3 enzymes in regulatory T cells, and Bai et al. [29] engineered bioactive surfaces to recruit endogenous BMSCs and enhance osteointegration. Han et al. [30] revealed that micropatterned surfaces modulate fibroblast‐to‐myofibroblast differentiation through the actin‐MRTF axis. These studies highlight the diversity of regulatory mechanisms in stem cell fate and tissue repair. In contrast, this research focuses on a direct, post‐translational interaction—the ubiquitination of a pseudokinase switch (TRIB3) by an E3 ligase (SIAH1)—and demonstrates that this axis is dysregulated in age‐related OP. By establishing a clear mechanistic link from SIAH1 to TRIB3 degradation and altered BMSC differentiation, we provide a new theoretical framework and a potential therapeutic target for OP. In this study, we provide multiple lines of conceptual advancement. First, we demonstrate that SIAH1‐mediated TRIB3 ubiquitination acts as a molecular switch controlling BMSC differentiation toward osteogenesis versus adipogenesis. This finding extends the known function of the SIAH1–TRIB3 axis beyond protein turnover in tumor cells to a physiologically relevant context of bone homeostasis. Second, by combining in vivo validation in naturally aged osteoporotic mice (including intrafemoral lentiviral knockdown of SIAH1) and serum ELISA analysis in osteoporotic patients, we provide the first cross‐species evidence that elevated SIAH1 and reduced TRIB3 levels are consistently associated with OP. Third, the TRIB3 rescue experiments unequivocally show that the effects of SIAH1 on osteogenic/adipogenic markers are specifically mediated through TRIB3, ruling out off‐target actions.

Subsequent in vivo and in vitro experiments confirmed that SIAH1 influences TRIB3 protein levels; SIAH1 overexpression significantly reduced TRIB3 expression, whereas its knockdown had the opposite effect. These results align with findings by Zhou et al. [25], Miyoshi et al. [31] and Hua et al. [32] who demonstrated that SIAH1 promotes TRIB3 degradation via ubiquitination in colorectal cancer stem cells and HEK 293T cells. The binding of β‐catenin to TRIB3 helps maintain TRIB3 protein homeostasis by competitively inhibiting SIAH1‐mediated ubiquitination and degradation [32]. Research indicates β‐catenin stabilizes TRIB3 by forming a complex with it [33, 34]. Inhibiting SIAH1 activity or expression significantly enhances TRIB3 protein stability. We found that in hBMSCs, SIAH1 modulates TRIB3 protein levels through the ubiquitin‐proteasome pathway and also influences markers such as ALP, RUNX2, and PPARγ [35]. Furthermore, SIAH1 and SIAH2 can mediate the ubiquitination of Smad proteins involved in bone metabolism and PPARγ, a key adipogenic regulator, thereby affecting lipid droplet formation [36–38]. Given TRIB3’s role as a key regulator of osteogenic/adipogenic differentiation, SIAH1 may influence related signaling pathways by controlling TRIB3 ubiquitination. For instance, SIAH1 transfection reverses the enhancing effect of TRIB3 on the TGF‐β pathway, and SIAH1‐mediated degradation of the TGF‐β effector TIEG1 restricts its regulatory function [25, 39].

While our MG132 experiments confirm the involvement of the ubiquitin‐proteasome system in SIAH1‐mediated TRIB3 degradation, we cannot exclude the possibility that the lysosomal‐autophagic pathway also contributes to this process. Recent studies have highlighted the intricate crosstalk between these two degradation systems. For instance, Wang et al. [40] demonstrated that ubiquitination can also act as a signal for selective autophagy, as seen with USP18 targeting GSDMD for autophagic degradation. Although our current study focuses on the proteasomal pathway, future investigations are warranted to determine whether TRIB3 is also subject to autophagic clearance, especially given that other post‐translational modifications like succinylation can influence ubiquitination and stability [41]. We have now included this important point as a direction for future research.

The functional outcome of ubiquitination is dictated by the topology of the ubiquitin chains. Our data, including the shortened half‐life of TRIB3 and the reversal of its degradation by MG132, strongly suggest that SIAH1 primarily conjugates TRIB3 with K48‐linked polyubiquitin chains, targeting it for proteasomal degradation. While other linkage types, such as K63 chains, are often involved in nonproteolytic signaling cascades [42], they could also be relevant in different contexts [43]. The specific linkage determination is a critical mechanistic question; future studies employing ubiquitin linkage‐specific antibodies or mass spectrometry will be essential to definitively map the chain types generated by SIAH1 on TRIB3 [42, 44, 45].

This study demonstrates that the SIAH1 ubiquitin ligase influences TRIB3 ubiquitination, but several limitations must be acknowledged. First, constraints in experimental funding precluded gene knockout studies in mice, which limited the depth of our in vivo analysis [46, 47]. Second, the cell lines used may not fully replicate the complex regulatory networks present in physiological environments, potentially affecting the extrapolation of results. We recognize that the mechanistic studies were performed in hBMSCs rather than primary mouse BMSCs. Although the SIAH1–TRIB3 ubiquitination pathway is highly conserved across mammals, further validation in mouse BMSCs would strengthen the translational relevance of our findings. Future studies will aim to replicate the key overexpression and knockdown experiments in primary mouse BMSCs to directly correlate with the mouse OP phenotype. Third, the precise structural domains mediating the SIAH1–TRIB3 interaction remain undefined, hindering a mechanistic understanding of the ubiquitination process. Finally, the dual regulatory role of SIAH1 in the osteogenic/adipogenic differentiation of BMSCs is not fully elucidated, which may impact the interpretation of our findings.

In summary, this study systematically reveals the effect of the SIAH1 ubiquitin ligase on the TRIB3 protein. Through bioinformatics analysis and experimental validation, we demonstrate that SIAH1 can effectively interfere with TRIB3 ubiquitination, affecting bone microstructure and related osteogenic/adipogenic proteins, thereby potentially contributing to the treatment of OP. This work provides a reliable foundation and a robust model for exploring these mechanisms in future clinical research.

## Author Contributions

Hao Lv, Hairu Yi, and Yan Wang designed the study, analyzed the data, and wrote the manuscript. Yinghao Zhu, Yuxiang Yang, Xingyu Wang, and Long Liang summarize and analyzed the data. Jiuxiang Wang and Ting Jiang revised the manuscript.

## Acknowledgments

The authors have nothing to report.

## Funding

This study was funded by the Anhui Provincial Natural Science Foundation (Grants 2408085MH226 and 2308085MH294), the Anhui University of Chinese Medicine Exploratory Research Projects (Grant AHUCM2024TS090), the Anhui Provincial Key Laboratory of Chinese Medicinal Formula (Grant 2025AKLCMF08).

## Disclosure

All authors have read and approved the final manuscript.

## Ethics Statement

All animal experiments were approved by the Animal Ethics Committee of the First Affiliated Hospital of Anhui University of Chinese Medicine, under Ethics Application Number AZYFY‐2024‐2006, which complied with the Ministry of Health’s Detailed Rules for the Implementation of the Administration of Medical Laboratory Animals, State Science and Technology Commission’s Regulations on the Administration of Laboratory Animals, ARRIVE guidelines, and the AVMA euthanasia guidelines 2020.

## Conflicts of Interest

The authors declare no conflicts of interest.

## Supporting Information

Additional supporting information can be found online in the Supporting Information section. Two supporting files were provided to support the data and experimental validation of this study, and relevant in‐text citations for these materials were integrated in the Section 3.

## Supporting information


**Supporting Information 1** Supporting Information File 1. This dataset contains full quantitative protein–protein docking energy values of TRIB3 with six candidate E3 ubiquitin ligases (SIAH1, NEDD4L, STUB1, SMURF1, HECW2, and WWP1) screened from bioinformatics analysis. The docking affinity scores quantitatively verified that SIAH1 possesses the strongest binding capacity toward TRIB3, which provided critical basis for selecting SIAH1 as the research object for subsequent mechanistic experiments. This supporting file was referenced in Section 3.1 (Bioinformatics Screening), when analyzing molecular docking results of candidate E3 ligases.

**Supporting Information 2** Supporting Information File 2. This supporting information presents flow cytometry identification data of passage 4 human bone marrow mesenchymal stem cells (hBMSCs). The detection results of surface markers CD34 and CD90 confirmed the mesenchymal stem cell phenotype of isolated primary cells, guaranteeing the reliability of all in vitro differentiation functional experiments. This dataset was cited in Section 3.4.1, when identifying the cell phenotype of hBMSCs before transfection and induction experiments.

## Data Availability

All raw data and code are available upon request.
