# Supplementary material for: SIAH1‐Mediated Ubiquitination and Degradation of TRIB3 Modulate Osteogenic and Adipogenic Differentiation
Source: Stem Cells Int. 2026 Aug 27;2026:5507620. doi: 10.1155/sci/5507620 (PMC13519890; doi:10.1155/sci/5507620)
Supplement: Supplementary file 1 [file SCI-2026-5507620-s001.pdf]

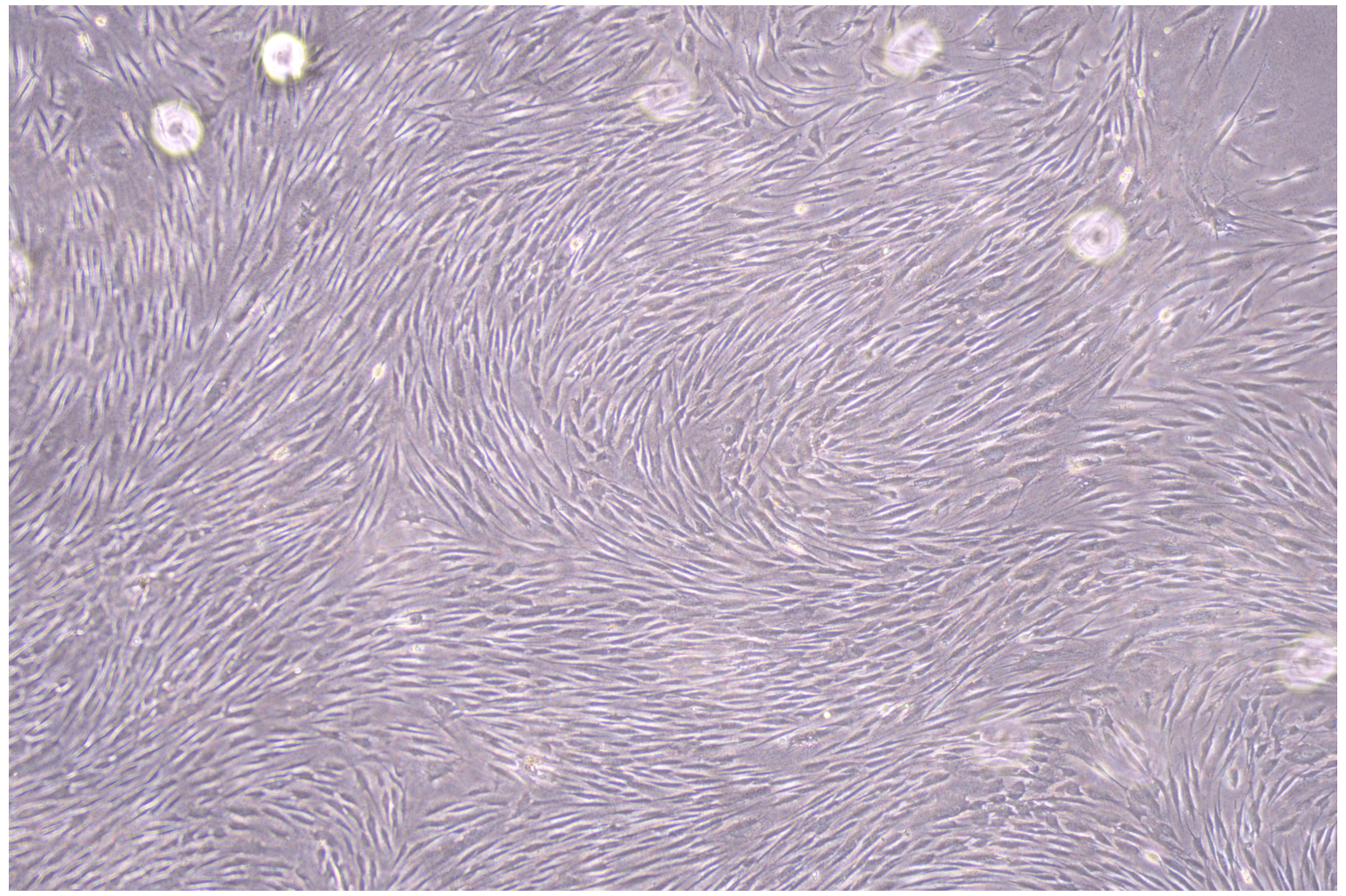

A

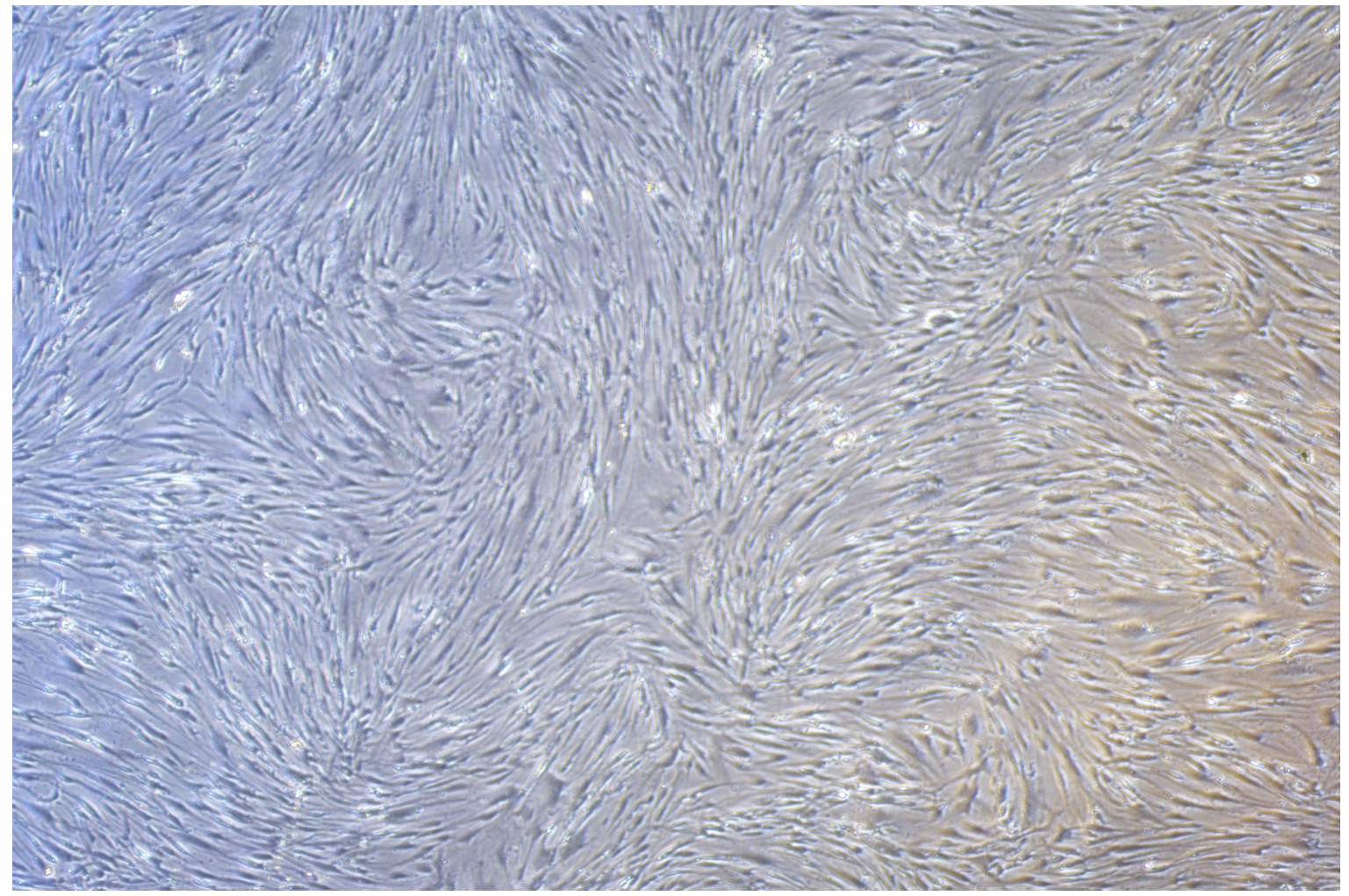

B

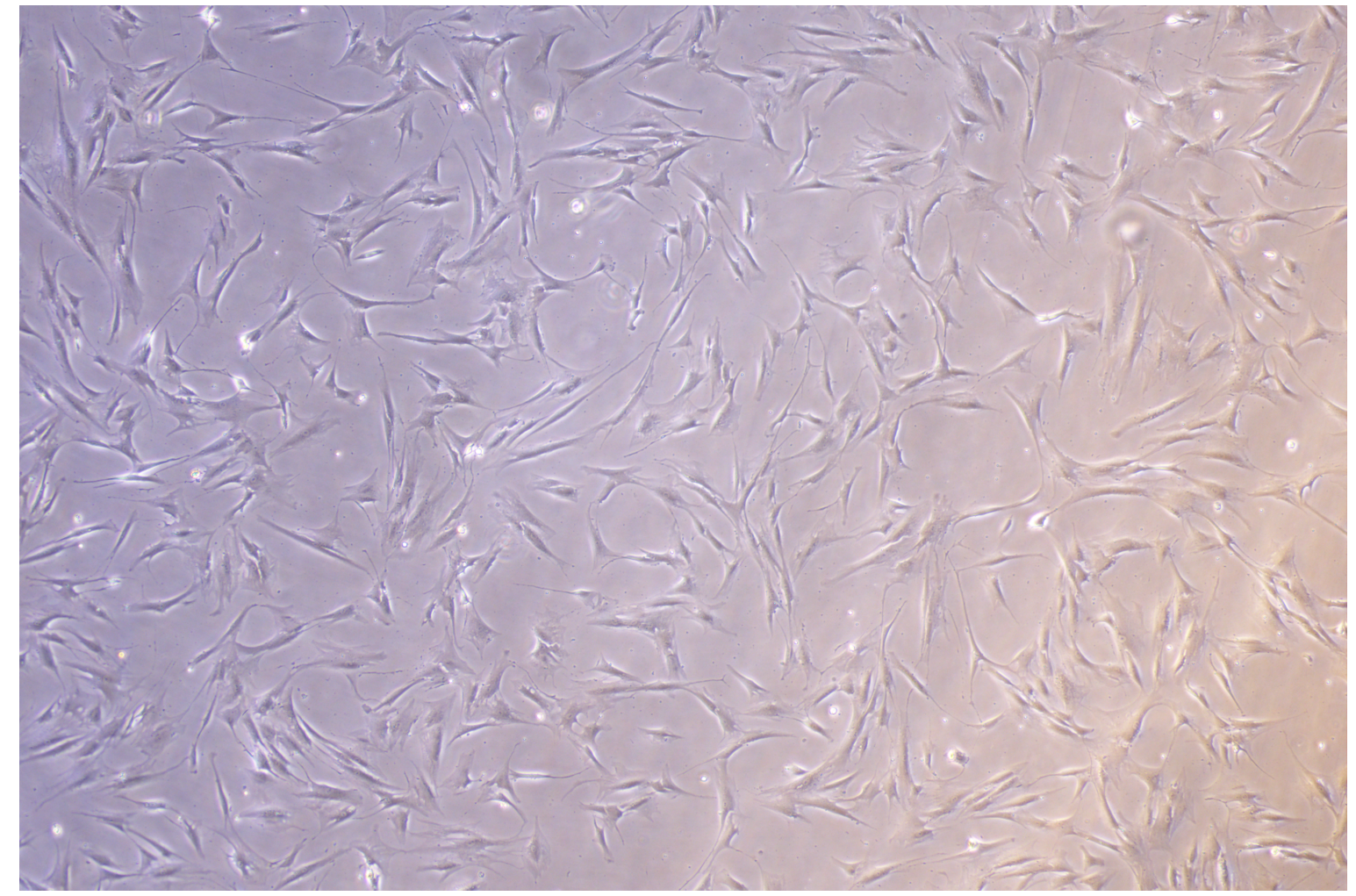

C

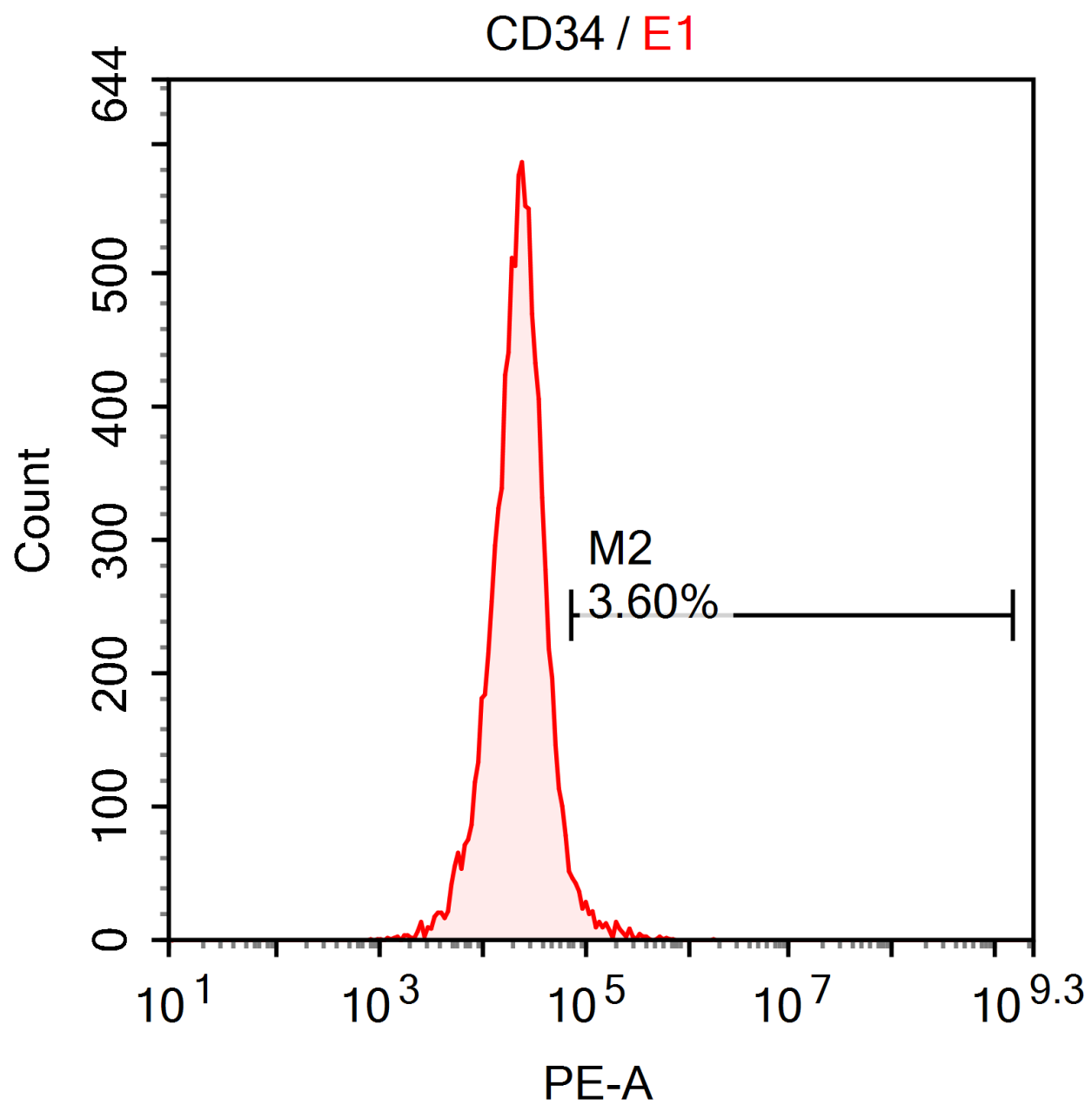
CD34 / E1
Count
0
100
200
300
400
500
644
M2
3.60%
10 1
10 3
10 5
10 7
10 9.3
PE-A

D

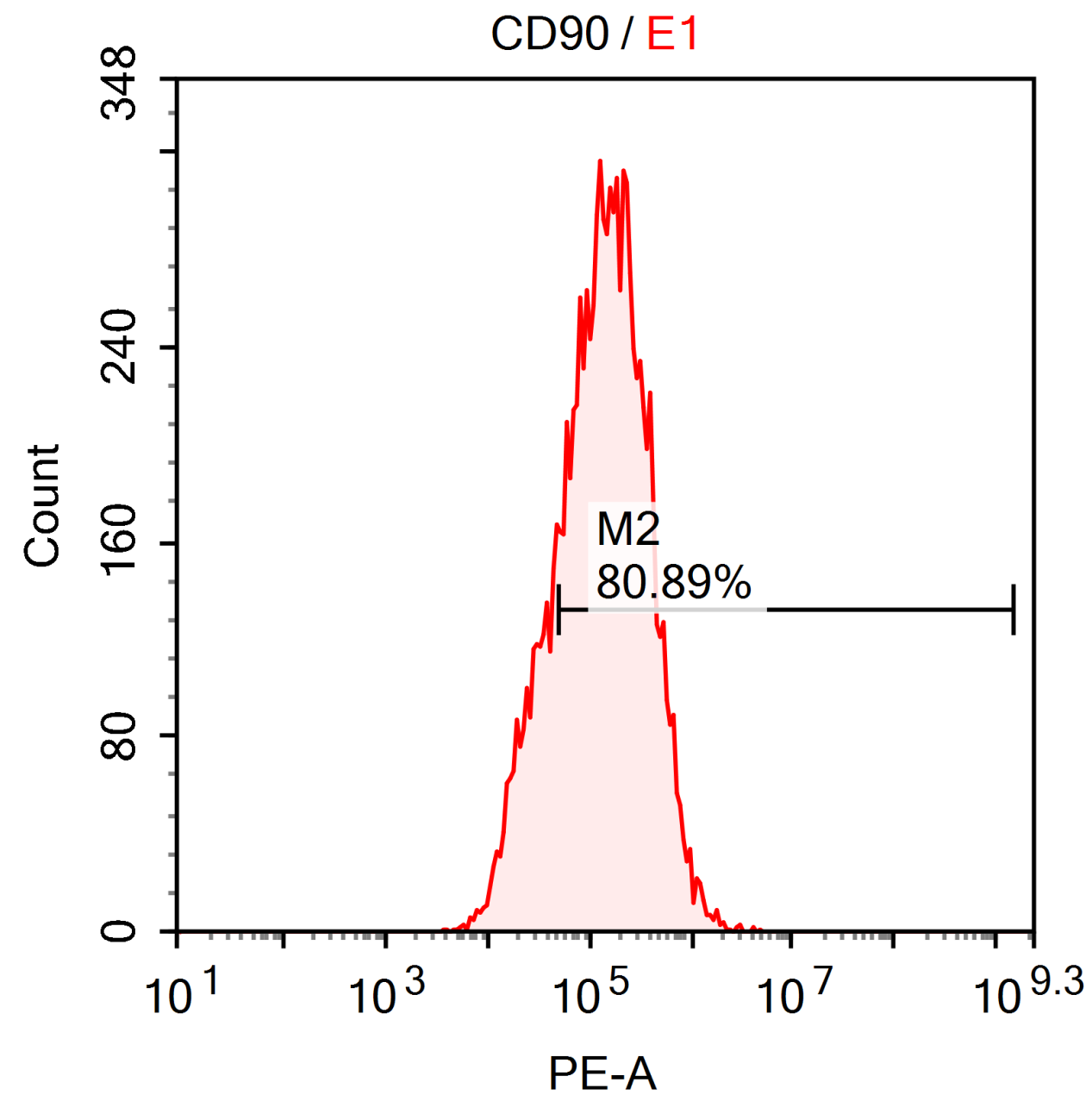
CD90 / E1
Count
0
80
160
240
348
M2
80.89%
10 1
10 3
10 5
10 7
10 9.3
PE-A

E
