# Supplementary material for: SIAH1‐Mediated Ubiquitination and Degradation of TRIB3 Modulate Osteogenic and Adipogenic Differentiation
Source: Stem Cells Int. 2026 Aug 27;2026:5507620. doi: 10.1155/sci/5507620 (PMC13519890; doi:10.1155/sci/5507620)
Supplement: Supplementary file 2 [file SCI-2026-5507620-s002.docx]

| **Dock protein** | **HDOCK_Score** | **RMSD** | **Rotation_Parameters_X** | **Rotation_Parameters_Y** | **Rotation_Parameters_Z** | **Translation_X** | **Translation_Y** | **Translation_Z** |
| --- | --- | --- | --- | --- | --- | --- | --- | --- |
| SIAH1-TRIB3 | -571.5 | 47.74 | 0.5111 | 1.67621 | -0.12915 | -571.5 | 47.74 | 1 |
| HECW-TRIB3 | -571.33 | 47.24 | 2.14649 | 1.96991 | 2.81006 | -571.33 | 47.24 | 1 |
| NEDD4L-TRIB3 | -559.41 | 28.96 | 6.0573 | 1.62721 | 0.77979 | -559.41 | 28.96 | 1 |
| SMURF1-TRIB3 | -571.22 | 57.17 | 1.07763 | 1.03594 | 0.79861 | -571.22 | 57.17 | 1 |
| STUB-TRIB3 | -568.02 | 32.52 | 3.48963 | 2.05232 | 0.14118 | -568.02 | 32.52 | 1 |
| WWP-TRIB3 | -563.5 | 49.41 | 5.52854 | 1.45183 | 0.64087 | -563.5 | 49.41 | 1 |
